# Mutated Leguminous Lectin Containing a Heparin-Binding like Motif in a Carbohydrate-Binding Loop Specifically Binds to Heparin

**DOI:** 10.1371/journal.pone.0145834

**Published:** 2015-12-29

**Authors:** Hirohito Abo, Keisuke Soga, Atsuhiro Tanaka, Hiroaki Tateno, Jun Hirabayashi, Kazuo Yamamoto

**Affiliations:** 1 Department of Integrated Biosciences, Graduate School of Frontier Sciences, The University of Tokyo, Kashiwa, 277–8562 Chiba, Japan; 2 Research Center for Stem Cell Engineering, National Institute of Advanced Industrial Science and Technology (AIST), Tsukuba, Ibaraki 305–8568, Japan; Consejo Superior de Investigaciones Cientificas, SPAIN

## Abstract

We previously introduced random mutations in the sugar-binding loops of a leguminous lectin and screened the resulting mutated lectins for novel specificities using cell surface display. Screening of a mutated peanut agglutinin (PNA), revealed a mutated PNA with a distinct preference for heparin. Glycan microarray analyses using the mutated lectin fused to the Fc region of human immunoglobulin, revealed that a particular sulfated glycosaminoglycan (GAG), heparin, had the highest binding affinity for mutated PNA among 97 glycans tested, although wild-type PNA showed affinity towards Galβ1-3GalNAc and similar galactosylated glycans. Further analyses of binding specificity using an enzyme-linked immunoadsorbent assay demonstrated that the mutated PNA specifically binds to heparin, and weakly to de-2-*O*-sulfated heparin, but not to other GAG chains including de-6-*O*-sulfated and de-*N*-sulfated heparins. The mutated PNA had six amino acid substitutions within the eight amino acid-long sugar-binding loop. In this loop, the heparin-binding like motif comprised three arginine residues at positions 124, 128, and 129, and a histidine at position 125 was present. Substitution of each arginine or histidine residue to alanine reduced heparin-binding ability, indicating that all of these basic amino acid residues contributed to heparin binding. Inhibition assay demonstrated that heparin and dextran sulfate strongly inhibited mutated PNA binding to heparin in dose-dependent manner. The mutated PNA could distinguish between CHO cells and proteoglycan-deficient mutant cells. This is the first report establishing a novel leguminous lectin that preferentially binds to highly sulfated heparin and may provide novel GAG-binding probes to distinguish between heterogeneous GAG repeating units.

## Introduction

Leguminous lectins represent the largest and most thoroughly studied lectin family with more than 100 members characterized. They consist of two or four identical subunits, each approximately 30 kDa in size and containing a single carbohydrate-binding site, also termed monosaccharide-binding site [1]. The carbohydrate-binding site of leguminous lectins consists of four loops (A-D), among which the long loop C is the main contributor to sugar-binding specificity and calcium binding [2]. The carbohydrate-binding specificity of lectins was classically divided into four classes based on the configuration at C-3 and C-4 of the lectin-reactive monosaccharide ring and may be alternatively classified into mannose/glucose-binding lectins (Makela's group III), galactose/*N*-acetylgalactosamine-binding lectins (Makela's group II), *N*-acetylglucosamine-binding lectins, fucose-binding lectins (Makela's group I), sialic acid-binding lectins, and those with complex binding sites [3]. The length of loop C in galactose/*N*-acetylgalactosamine-binding, mannose/glucose-binding, *N*-acetylglucosamine-binding, and sialic acid-binding leguminous lectins is eight, eight, ten, and ten residues, respectively [2,4,5]. Thus lectin specificity for larger sugar units appears to correlate with a longer length of loop C.

Recently, we reported a novel mammalian cell surface display method for generating mutated lectins with novel sugar-binding specificities [6]. To evaluate the relationship between amino acid sequence, especially in the region of the carbohydrate-binding loops, and the sugar-binding specificity of leguminous lectins, we introduced random mutations in the carbohydrate-binding loops of peanut agglutinin (PNA), expressed on the surface of mammalian cells, and successfully screened for mutated lectins specific for NeuAcα2-6(Galβ1–3)GalNAc [6]. We observed that the carbohydrate-binding loop C was critically involved in determining lectin specificity and identified several critical amino acid residues within this loop associated with sugar-binding activity and/or sugar-binding specificity. Data obtained by X-ray crystallographic analyses of many leguminous lectin-oligosaccharide complexes also indicate that amino acid residues within loop C are largely involved in both lectin binding and specificity via hydrophilic and hydrophobic interaction with two to three sugar units [1]. With few exceptions, leguminous lectins interact with the non-reducing, terminal sugar residues of oligosaccharides and polysaccharides at a primary "monosaccharide-binding site" localized at the bottom of the carbohydrate-binding site. Glycosaminoglycan (GAG)-binding proteins bind to sugar residues that lie within a disaccharide-repeating unit consisting of uronic acid and an amino sugar instead of at the terminus. This binding mode of GAG-binding proteins is distinct from that of leguminous lectins and could explain why leguminous lectins with binding specificity for GAGs have not yet been reported.

Heparan sulfate proteoglycans/heparin play a pivotal role in many biological processes such as cell adhesion, cell growth, cell motility and tumor formation [7], and the GAG moiety on proteoglycans is essential for these functions. Located on the extracellular matrix and cell surfaces, heparan sulfate proteoglycans act as co-receptors regulating the local retention and stabilization of heparin-binding growth factors. During inflammation, for example, chemokines selectively recruit leukocytes in a process that requires immobilization of chemokines on heparan sulfate proteoglycans in the extracellular matrix. The interaction of basic growth factors and chemokines with GAGs is not simply the result of global electrostatic attraction between these proteins and acidic GAGs. Cardin and Weintraub analyzed the consensus sequences of 49 regions in 21 heparin-biding proteins based on the sequence organization of their basic and non-basic amino acid residues and identified as common heparin-binding motifs the XBBXBX and XBBBXXBX sequences, where B is a Lys or Arg (rarely His) and X, a hydropathic residue such as Ala, Gly, Ile, Leu or Tyr [8]. Torrent *et al*. reported that CPC clip motifs in heparin-binding proteins, where C is a cationic residue and P is a polar residue, are also involved in binding to heparin and other sulfated GAGs [9].

Here we report a novel mutated PNA lectin, generated by the screening of a mutated PNA library, that specifically binds to heparin. The lectin contains a heparin-binding like motif involving six amino acid substitutions in the carbohydrate-binding loop C. These substitutions were sufficient to switch the carbohydrate-binding specificity of PNA from Galβ1-3GalNAc to highly sulfated heparin. Inhibition assay demonstrated that dextran sulfate inhibited mutated PNA binding to heparin at lower concentrations that heparin. It is hoped that this work will help guide the engineering of novel glycan probes, thereby furthering the study of the significance and function of GAGs.

## Materials and Methods

### Cells and reagents

A human T cell hybridoma (2B4) harboring a green fluorescent protein (GFP)-reporter gene under the control of the nuclear factor of activated T cells (NF-AT) was kindly provided by Dr. H. Arase (Osaka University, Osaka, Japan) [10]. The retroviral vector, pMXs vector, and Plat-E retrovirus-packaging cells were provided by Dr. T. Kitamura (The Institute of Medical Sciences, The University of Tokyo, Tokyo, Japan) and were used for retrovirus transduction throughout this study [11]. CHO cells and their proteoglycan-deficient mutants, PgsA-745, PgsB-618, PgsD-677, and PgsE-606 were obtained from the American Type Culture Collection (Manassas, VA, USA). HEK293 cells were obtained from the Cell Resource Center for Biomedical Research (Tohoku University, Miyagi, Japan). Plat-E cells were maintained in DMEM medium (Invitrogen, Carlsbad, CA, USA) supplemented with 25 mM 4-(2-hydroxyethyl)-1-piperazineethanesulfonic acid (HEPES) (pH 7.4), 2 mM glutamine, 10 μg/ml blasticidin S (Invitrogen), and 1 μg/ml puromycin (Sigma-Aldrich, St. Louis, MO, USA). The remaining cells were cultured in DMEM medium supplemented with 10% heat-inactivated fetal bovine serum (FBS), 2 mM glutamine, and 25 mM HEPES (pH 7.4). All cell lines were cultured at 37°C under 5% CO_2_ conditions. Heparin from porcine intestinal mucosa was purchased from Merck (M.W. approx. 15,000, Darmstadt, Germany) and dextran sulfate was from Sigma-Aldrich (M.W. approx. 10,000). Chondroitin (CH) from shark cartilage, chondroitin sulfate A (CS-A) from whale cartilage, chondroitin sulfate B (CS-B) from pig skin, chondroitin sulfate C (CS-C) from shark cartilage, and heparan sulfate (HS) from bovine kidney were purchased from Seikagaku Kogyo (Tokyo, Japan). 2-*O*-Desulfated heparin (2-DS HP), 6-*O*-desulfated heparin (6-DS HP), *N*-desulfated heparin (*N*-DS HP) and *N*-desulfated, *N*-acetylated heparin (*N*-DS,Ac HP) were purchased from PG research (Tokyo, Japan). Hyaluronic acid (HA) from *Streptococcus zooepidemicus* was kindly provided by Shiseido Co. (Tokyo, Japan). Bovine serum albumin (BSA) and *N*-ethoxycarbonyl-2-ethoxy-1,2-dihydroquinoline (EEDQ) were purchased from Wako pure chemicals (Tokyo, Japan).

### Establishment of mutated PNA library-expressing reporter cells and screening of mutated lectins by a GFP-reporter assay

Recombinant DNA experiments were conducted in accordance with a comprehensive, high quality care program, which has been approved by the Life Science Research Committee of the Graduate School of Frontier Sciences of The University of Tokyo guided by the Life Science Committee of The University of Tokyo. Establishment of mutated PNA library-expressing reporter cells was described previously [6]. The 2B4 cells expressing mutated PNA on their cell surface were stained with an anti-myc antibody (9E10; American Type Culture Collection) and *R*-phycoerythrin (PE)-labeled goat anti-mouse IgG-F(ab')_2_ (Beckman Coulter, Fullerton, CA, USA), and then enriched using a FACS Vantage SE cell sorter (BD Biosciences, San Jose, CA, USA) based on the expression of the myc-tag. The enriched 2B4 cells expressing mutated PNA were cultured in 6-well ELISA plates (Iwaki, Tokyo, Japan) coated with heparin conjugated to BSA for 16 h at 37°C. A reporter assay of each cloned cell grown on various glycan-coated wells was performed using the FACS Calibur system (BD Biosciences), and data were analyzed using FlowJo (TreeStar, San Carlos, CA, USA).

### Isolation and sequencing of cloned mutated PNA cDNAs

Genomic DNA was extracted from the cloned GFP-positive cells using the FlexiGene DNA Kit (Qiagen) according to the manufacturer's protocol. The cDNAs encoding mutated lectins were then amplified by polymerase chain reaction (PCR) using a forward primer (5'-CGGAATTCGCCGAAACAGTTTCCTTCC-3') containing an EcoRI site and a reverse primer (5'-CCGCTCGAGTGCACTTGCCATATTCAT-3') containing an XhoI site, subcloned into the pBluescript II SK(+) vector, and sequenced using an ABI 3500/3500xL genetic analyzer (Applied Biosystems, Foster City, CA, USA). Each putative lectin cDNA was subcloned into pMXs-CD3ζ and expressed in 2B4 cells. Cloned lectin mutants expressed on the cell surface were subjected to GFP-reporter assays in several different glycan-coated wells, as described above.

### Preparation of mutated PNA-IgG Fc fusion proteins

A cloned lectin cDNA fused to the cDNA encoding the Fc segment of human IgG_1_ (provided by Dr. H. Arase, Osaka University) was inserted into the pCAGGS vector to generate pCAGGS-mPNA-Fc [12]. HEK293 cells were transfected with pCAGGS-mPNA-Fc using Lipofectamine 2000 reagent (Invitrogen), and cells stably secreting mutated PNA-Fc proteins were selected by culturing in medium containing 1 mg/ml G418 (Sigma-Aldrich). After dilution cloning of G418-resistant cells, the clone that displayed the highest production of the mutated PNA-Fc fusion protein was chosen and subjected to a large-scale culture. The mutated PNA-Fc fusion protein was purified from conditioned media of the cells by affinity chromatography using a HiTrap rProtein A FF column (GE Healthcare, Piscataway, NJ, USA) and the AKTA Explorer system (GE Healthcare). The purity was confirmed by SDS-PAGE on 10% acrylamide gel under reducing conditions. To introduce a mutation into the cDNAs encoding alanine-substituted, mutated PNA(H)-Fc clones, the KOD plus mutagenesis kit (Toyobo, Osaka, Japan) and the following primers were used according to the manufacturer’s protocol. R124A-F, 5′-ATGCTCATGTGAACAGGCGTTAT-3′; R124A-R, 5′-CAAACTCCACTCCAACAAAGTAACC-3′; H125A-F, 5′-GTGCTGTGAACAGGCGTTAT-3′; H125A-R, 5′-GATCAAACTCCACTCCAACAAA-3′; R128A-F, 5′-ACGCGCGTTATTCGGATCCTCC-3′; R128A-R, 5′-TCACATGACGATCAAACTCCACTCCA-3′; R129A-F, 5′-GGGCTTATTCGGATCCTCCCACT-3′; R19A-R, 5′-TGTTCACATGACGATCAAACTCCAC-3′.

### Preparation of GAG-conjugated BSA and binding of mutated PNA-IgG Fc fusion proteins to several GAG-conjugated BSA

Coupling of GAGs to BSA with the aid of EEDQ was performed according to the method of Ishitsuka *et al*. [13]. Briefly, 5.0 mg of GAG dissolved in 400 μl of distilled water were added to 500 μl of 10 mg/ml EEDQ in ethanol and incubated for 1 h at 25°C. After the reaction mixture was cooled at 4°C, 500 μl of 20 mg/ml BSA in distilled water was added and incubated for 12 h at 4°C. Finally the reaction solution was concentrated to 500 μl in Tris-buffered saline (TBS, 20 mM Tris-HCl, pH 8.0, and 150 mM NaCl) by ultrafiltration using an Amicon ultra-15 (Amicon, Beverly, MA). The concentration of GAG-BSA was assessed by BCA protein assay kit (Pierce, Rockford, IL). A 96-well enzyme-linked immunosorbent assay (ELISA) plate was coated with 30 μl of 30 μg/ml GAG-BSAs in TBS at 4°C for 16 h. After the plate was washed with PBS three times, and mutated PNA-Fc (final concentration 11.6 μg/ml) was added and incubated for 4 h at 20°C. The plate was washed with phosphate-buffered saline (PBS) twice, added with alkaline phosphatase-labeled anti-human IgG-Fc antibody, and incubated for 1 h at 20°C. After washing with PBS and incubated with 4.6 mM *p*-nitrophenyl phosphate (Sigma-Aldrich), alkaline phosphatase activity was measured as the absorbance at 405 nm. In case of binding inhibitory assay with GAGs and GAG-unrelated heparin analogs such as dextran sulfate (Sigma-Aldrich), deoxyribonucleic acid (Sigma-Aldrich), or phytic acid (Wako Chemicals, Tokyo, Japan), mutated PNA-Fc was preincubated with the indicated concentration of an inhibitor for 2 h at 4°C and then measured the binding of mutated PNA-Fc (10 μg/ml) to heparin-coupled BSA was performed as described above.

The binding of PNA-Fc and mutated PNA-Fc to Chinese hamster ovary (CHO) cell and its proteoglycan-deficient mutants, PgsA-745, PgsB-618, PgsD-677, or PgsE-606 cells, were monitored by measuring the mean fluorescent intensity (MFI) using flow cytometry as previously described [6].

### Glycan microarray

The sugar-binding specificities of the mutated PNA proteins were analyzed using a glycan microarray (version 4.2) [14]. Briefly, 100 μl of mutated PNA-Fc fusion protein (50 μg/ml) was loaded onto the glycan microarray and incubated at 20°C for 18 h. After washing with probing buffer (10 mM Tris-HCl (pH 7.4) containing 0.15 M NaCl and 0.02% Tween 20), 100 μl of cyanine dye 3 (Cy3)-labeled donkey anti-human IgG-Fcγ antibody (1 μg/ml; Jackson ImmunoResearch, West Grove, PA, USA) in probing buffer was applied to the microarray and incubated at 20°C for 3 h. After a further wash with probing buffer, the binding of mutated PNA-Fc fusion protein was detected using the SC-profiler evanescent field fluorescence-assisted scanner (GP Biosciences, Yokohama, Japan) in the Cy3 mode.

### SPR analysis of the interaction of mutated PNA-Fc with heparin

Surface plasmon resonance (SPR) experiment was performed with the Biacore X100 system and Biacore X100 evaluation software (Biacore AB, Uppsala, Sweden). Heparin was coupled with biotin-hydrazide (Dojindo, Kumamoto, Japan) by reductive amination. Briefly, 1 mg of heparin solubilized in 125 μl of 0.1 M 2-(*N*-morpholino)ethanesulfonic acid (MES), pH 5.5, was mixed with 250 μl of 72 mM biotin-hydrazide in dimethyl sulfoxide and 250 μl of 1 M sodium cyanoborohydride in 0.1 M MES, pH 5.5. After the mixture was allowed to stand at 20°C for 12 h, biotinylated heparin was purified on a column of Superdex peptide (3 × 250 mm, GE Healthcare). Biotinylated heparin was immobilized to streptavidin-coupled sensor chip SA (Biacore) until the resonance units reached approximately saturation (approximately 130 resonance unit (RU)). The binding of mutated PNA-Fc to heparin was measured in 10 mM HEPES, pH 7.4, containing 150 mM NaCl and 0.005% surfactant P20 (GE Healthcare) at 25°C with a flow rate of 20 μl/min. The mutated PNA-Fc at 1, 0.2, 0.04, 0.008, and 0.0016 μM was applied to the sensor chip. The regeneration of the chip surface was carried out with 50 mM glycine-HCl, pH 3.0, containing 150 mM NaCl and 0.005% surfactant P20.

## Results

### Identification of mutated PNA

**Fig 1:**
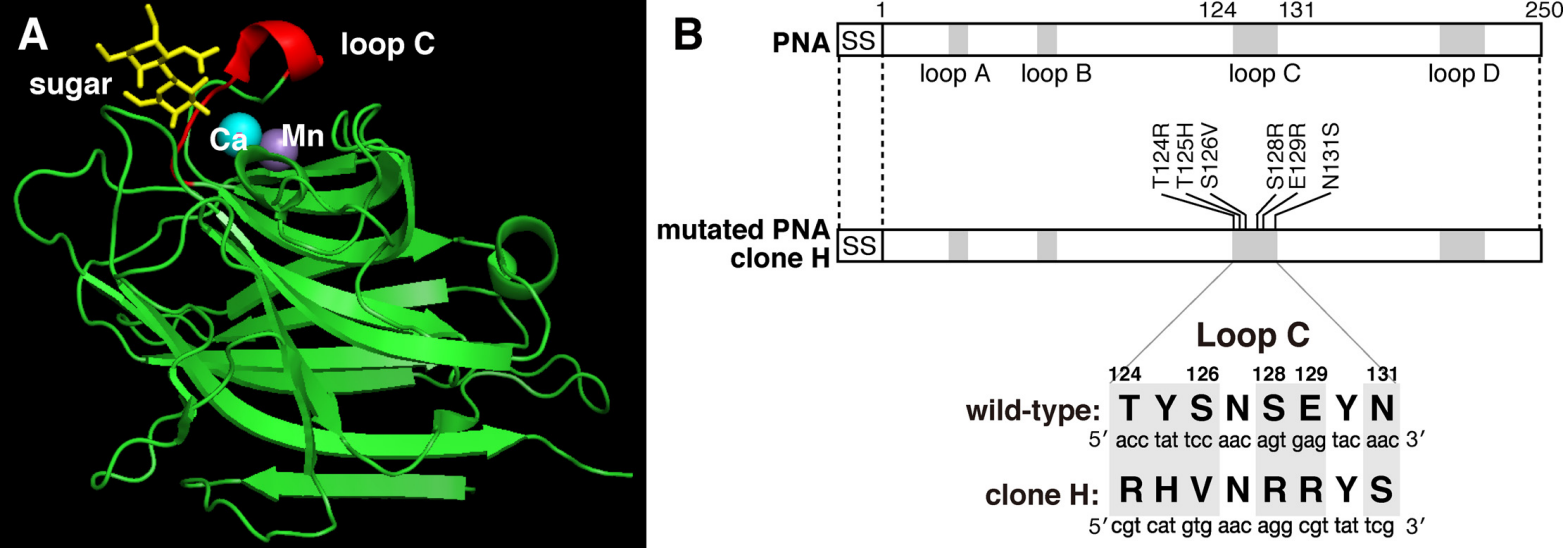
Tertiary structure of PNA and amino acid substitutions in mutated PNA clone H. (A) Schematic illustration of the three-dimensional structure of PNA (PDB entry 2DVA). Ribbon representation of the main chain of PNA (green). Loop C consisting of the sugar-binding site is shown in red. Calcium and manganese ions are indicated by balls colored in cyan and magenta, respectively, and the sugar ligand (Galβ1-3GalNAc) complexed with PNA is shown as yellow sticks. (B) Amino acid substitutions of mutated PNA clone H (mPNA(H)) compared with wild-type PNA. The sugar-binding site of PNA consists of four loops (A-D) with six amino acid substitutions in loop C of mPNA(H).

**Fig 2:**
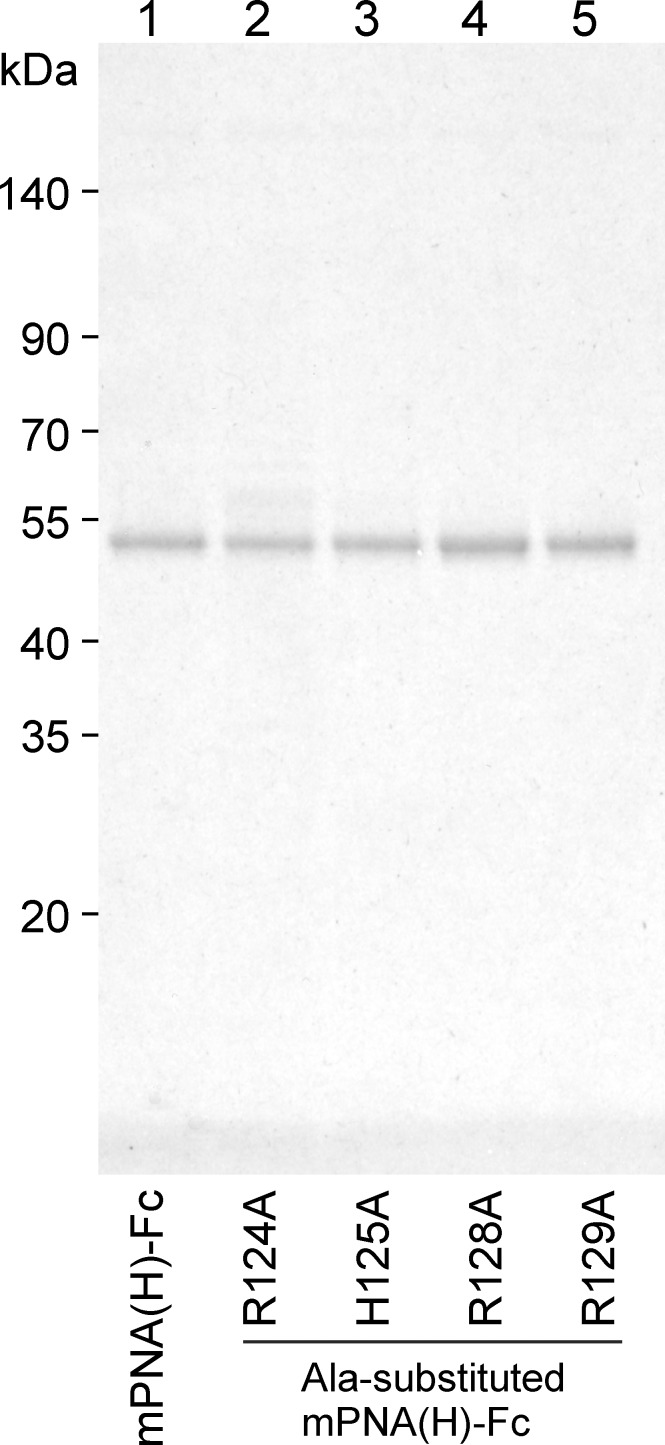
SDS-PAGE of the purified mutated PNA clone H-IgG Fc fusion protein and its Ala-substituted variants. mPNA(H)-Fc and Ala-substituted mPNA(H)-Fc at positions Arg124, His125, Arg128, or Arg129, respectively, were expressed, purified, and analyzed by electrophoresis on a 10% polyacrylamide gel under reduced conditions.

**Fig 3:**
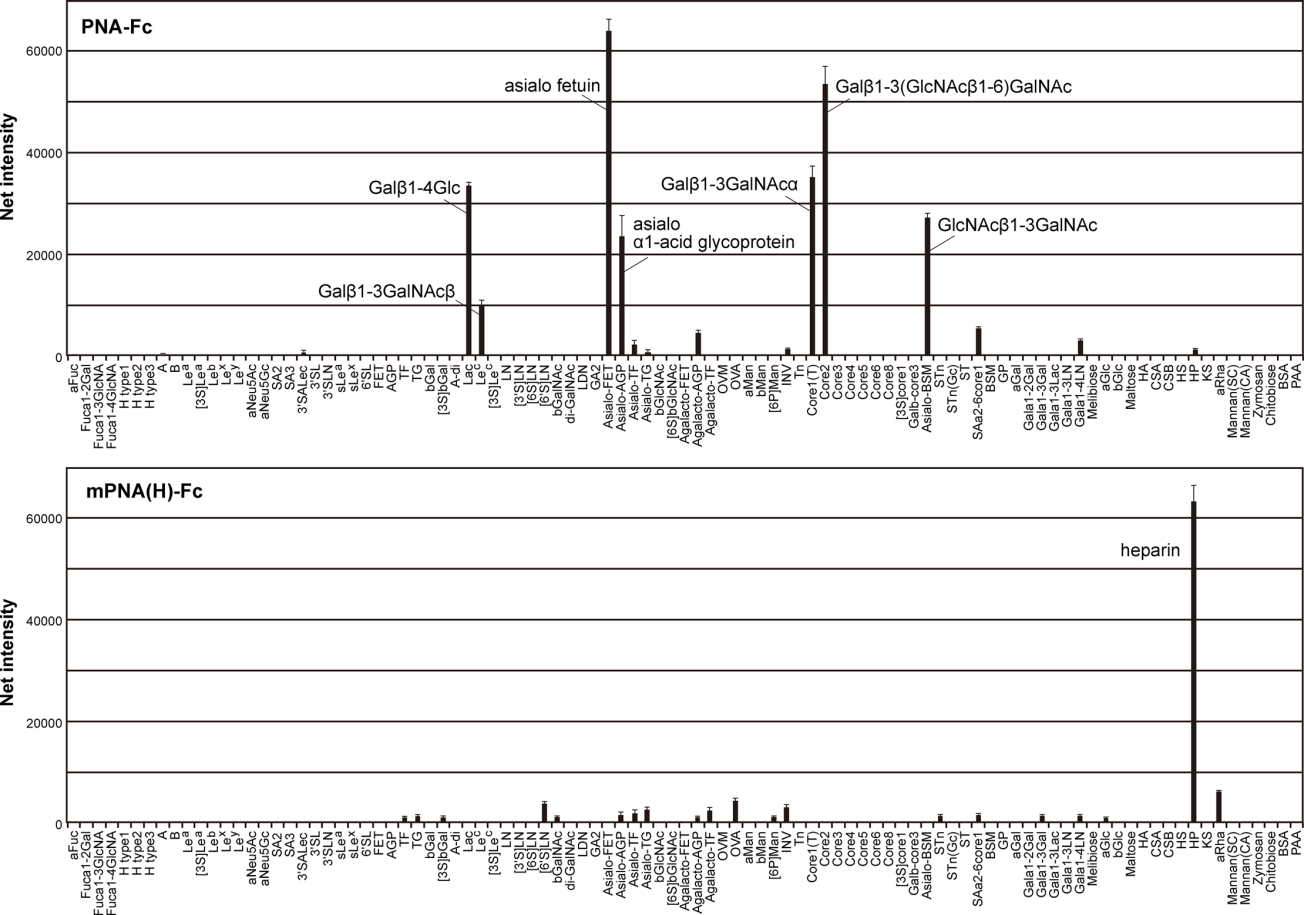
Glycan microarray analysis of wild-type and mPNA(H)-Fc. Oligosaccharides and glycoproteins used in this experiment (97 in total) are shown in S1 Fig. The data are represented as the mean ± SD of n = 3 independent spots.

**Fig 4:**
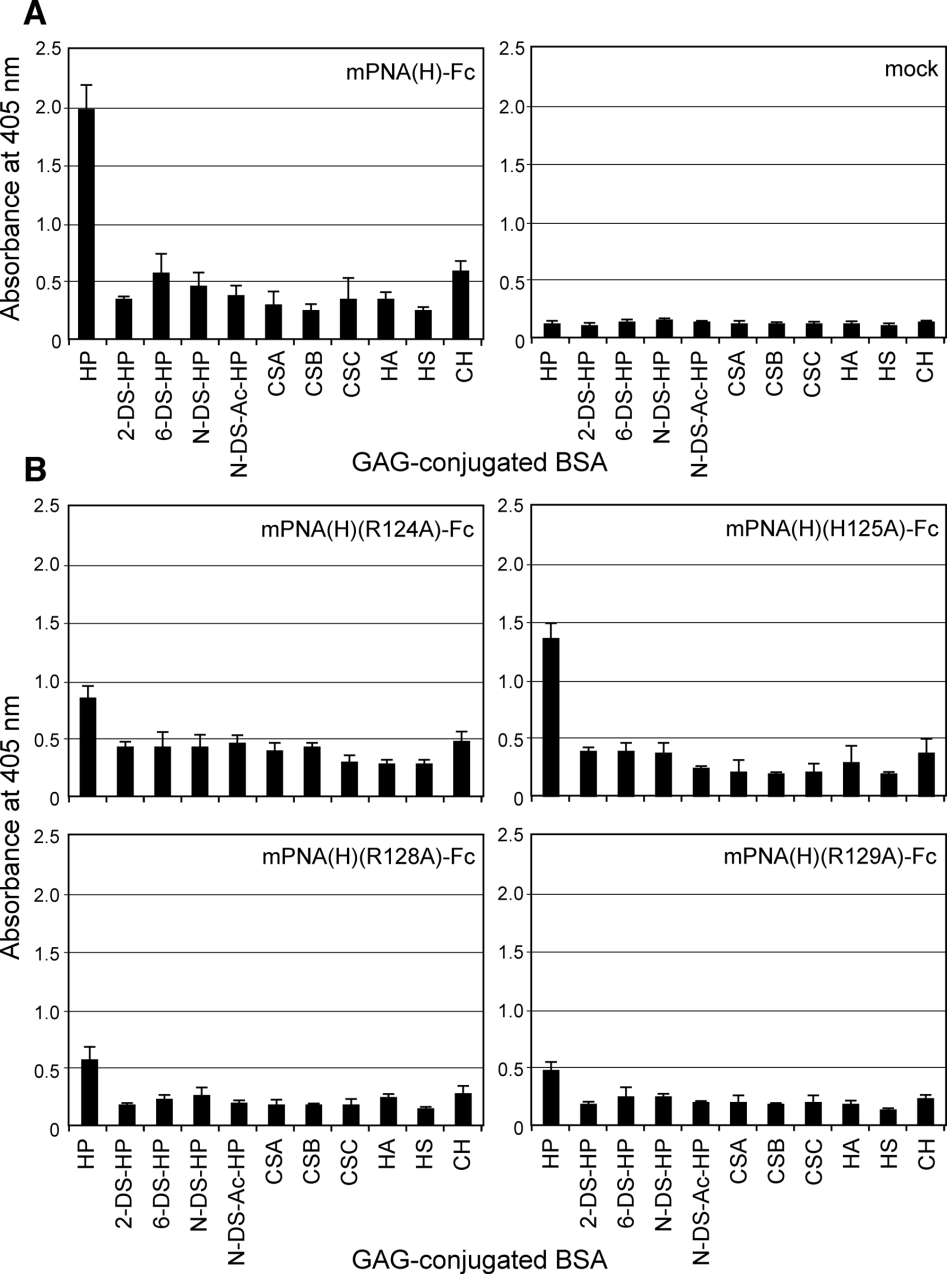
Binding of different glycosaminoglycan-conjugated BSAs to (A) mPNA(H)-Fc and IgG Fc (mock), and (B) mPNA(H)-Fc Ala-substituted at positions Arg124, His125, Arg128, or Arg129. The data are represented as the mean ± SD of n = 3 independent wells. HP, heparin; 2-DS-HP, 2-*O*-desulfated heparin; 6-DS-HP, 6-*O*-desulfated heparin; *N*-DS-HP, 2-*N*-desulfated heparin; *N*-DS-Ac-HP, 2-*N*-desulfated, acetylated heparin; CSA, chondroitin sulfate A; CSB, chondroitin sulfate B; CSC, chondroitin sulfate C; HA, hyaluronic acid; HS, heparan sulfate; CH, chondroitin.

In previous work, we established a method for the efficient expression of a leguminous lectin on the surface of mammalian cells (cell surface display method) without loss of sugar-binding ability. Furthermore, we prepared a mutated PNA library whose sugar-binding loops C and/or D, eight and seven amino acids in length respectively, were substituted with random amino acid residues. Since the carbohydrate-binding site of leguminous lectins consists of four loops (A-D) with loops C and D primarily involved in sugar-binding (Fig 1A) [2], the sugar-binding specificity of leguminous lectin was expected to be affected by the introduction of mutations in these loops. Mutated lectins that preferentially bound to NeuAcα2-6(Galβ1–3)GalNAc were cloned from mutated PNA library expressed on the cell surface [6]. Using these screening methods, a mutated PNA clone that bound to heparin (highly sulfated heparan sulfate) was isolated. Compared to wild-type PNA and out of a total of 250 amino acid residues, the mutated PNA clone H (mPNA(H)) had six substitutions in the coding region of loop C (Fig 1B). The mutated loop C amino acid sequence was RHVNRRYS whereas in wild-type PNA the sequence was TYSNSEYN. To test sugar-binding activity and specificity, mutated PNA fused to the Fc region of human IgG (mPNA(H)-Fc) was expressed in HEK293T cell and purified from the supernatant of cultured media of the cell (Fig 2, lane 1). mPNA(H)-Fc specifically binds to heparin whereas wild-type PNA binds to several galactosylated glycans and glycoproteins, but not to glycosaminoglycans including heparin (Fig 3). Binding of mPNA(H)-Fc to 2-*O*-desulfated heparin, 6-*O*-desulfated heparin or *N*-desulfated heparin was decreased to 18%, 29% or 23% of its binding to heparin, respectively (Fig 4A). These results indicate that 2-*O*-, 6-*O*-, and *N*-sulfations were required for high affinity binding of mPNA(H)-Fc to heparin.

### Involvement of a heparin-binding like motif of mutated PNA(H) in heparin binding

mPNA(H) contained a heparin-binding like motif in loop C of type BBXXBBXX where B is a Lys or Arg (rarely His), which is similar to a common heparin-binding motif [8]. A strictly conserved amino acid, Asn127, and Tyr130, a highly conserved residue among leguminous lectins, were also present (Fig 1B). To test whether the putative heparin-binding motif of mPNA(H) was involved in heparin binding, mPNA(H)-Fc proteins with Ala substitutions in residues 124, 125, 128, or 129 were expressed in HEK293T cells and purified (Fig 2, lanes 2–5), and their binding to several GAGs was measured by ELISA. Substitution of Arg124 to Ala reduced binding to 43% of the value of unsubstituted mPNA(H)-Fc (Fig 4B). His125 involvement was moderate with H125A mPNA(H)-Fc affinity for heparin maintained at approximately 70% of the unsubstituted mPNA(H)-Fc. By contrast, both Arg128 and Arg129 were critically involved in heparin binding with the affinity of mPNA(H)-Fc dropping to less than 30% of the unsubstituted mPNA(H)-Fc value when either Arg128 or Arg129 were substituted to Ala. Binding of Ala-substituted mPNA(H)-Fc proteins to other GAG chains was not affected (Fig 4B), indicating that the heparin-binding like motif of mPNA(H) is specific to heparin but not to other glycosaminoglycan chains.

**Fig 5:**
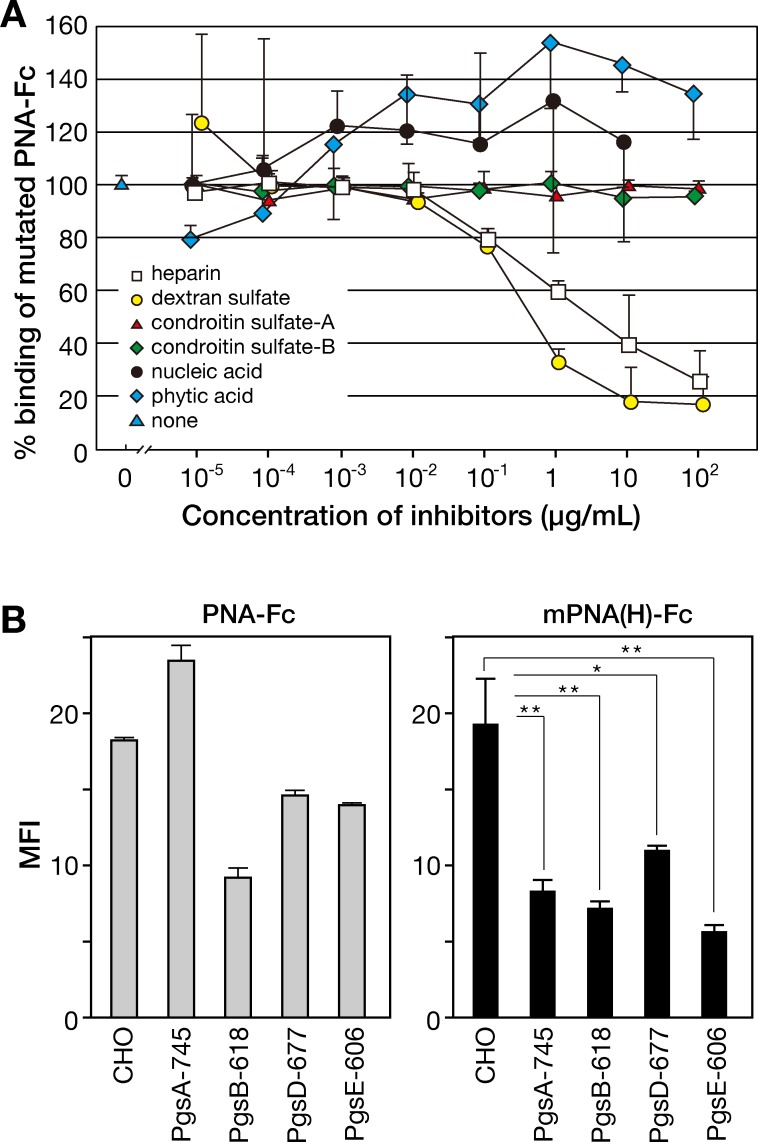
Binding inhibition assay and binding of PNA-Fc and mPNA(H)-Fc to CHO or its proteoglycan-deficient mutants using flow cytometry. (A) Binding inhibition assay using heparin (white rectangle), chondroitin sulfate-A (red triangle), chondroitin sulfate-B (green rectangle) and GAG-unrelated heparin analogs, dextran sulfate (yellow circle), deoxyribonucleic acid (black circle), or phytic acid (blue square). The mutated PNA-Fc was preincubated with the indicated concentration of an inhibitor for 2 h at 4°C and then measured the binding of mutated PNA-Fc to heparin-coupled BSA was performed as described in Materials and Methods section. The binding of mPNA(H)-Fc in the absence of competitor was to be 100% (blue triangle). (B) Cells stained with wild-type PNA-Fc (left) or mPNA(H)-Fc (right) were analyzed by flow cytometry and the binding of mPNA(H)-Fc to the cells was monitored by measuring the mean fluorescent intensity (MFI). The data are represented as the mean ± SD of n = 3 independent experiments. * *p*<0.05, ** *p*<0.01 (one-way factorial analysis of variance).

To get a deeper insight into the specificity of the mutant PNA-heparin interaction, inhibition assay of mutated PNA-Fc binding to heparin in the presence of putative inhibitors to determine minimal concentrations of the competitors to avoid mutated PNA-Fc binding. As shown in Fig 5A, heparin and dextran sulfate strongly inhibited mPNA(H)-binding in dose-dependent manner, and IC_50_ values of heparin and dextran sulfate were approximately 4.4 x 10^−7^ and 8.3 x 10^−8^ M, respectively. By contrast, chondroitin sulfate-A and -B, deoxyribonucleic acid and phytic acid did not abrogate the interaction between mPNA(H)-Fc and heparin, though these glycosaminoglycans and polyanionic compounds are also highly acidic. Further, we measured the dissociation constant (*K*_*d*_) value of the interaction between mPNA(H)-Fc and heparin by using surface plasmon resonance. Biotinylated heparin was immobilized on sensor chip and several concentrations of mPNA(H)-Fc were eluted. Based on the obtained data, *K*_*d*_ value of the interaction between mPNA(H)-Fc and heparin was 2.47 x 10^−8^ M (Table 1). Given that mPNA(H)-Fc bound to heparin with high affinity, partially desulfated heparins such as 2-*O*-desulfated heparin, 6-*O*-desulfated heparin or *N*-desulfated heparin (Fig 4A) might be still be good ligands for mutated PNA.

**Table 1:** Kinetics parameters of the interaction between mutated PNA-Fc and biotinylated heparin obtained from SPR analysis using Biacore

	*k*_*a*_ [1/Ms]	*k*_*d*_ [1/s]	*K*_*d*_ [M]	*K*_*d*_ [M] (mean ± SD)
**1**	2.58 x 10^4^	6.34 x 10^−4^	2.46 x 10^−8^	
**2**	2.27 x 10^4^	6.82 x 10^−4^	3.01 x 10^−8^	2.47±0.54 x 10^−8^ [M]
**3**	2.66 x 10^4^	5.18 x 10^−4^	1.94 x 10^−8^	(n = 3)

Using flow cytometry, we measured the binding of mPNA(H)-Fc to CHO cells and their proteoglycan-deficient mutant cells, PgsA-745, PgsB-618, PgsD-677, and PgsE-606, which are deficient in xylosyltransferase I, galactosyltransfease I, *N*-acetylglucosaminyl- and glucuronosyltransferase, and *N*-sulfotransferase genes, respectively [7,15–17]. PgsA-745 and PgsB-618 cells have defects in the initiation of glycosaminoglycan synthesis, and PgsD-677 cells have a defect in the extension step of the repeating disaccharide unit of heparin/heparan sulfate [18]. In PgsE-606 cells, *N*-sulfation of heparin/heparan sulfate is reduced. Wild-type PNA-Fc bound to the surface of these cells, however, MFI values of PNA-Fc binding was not correlated with the decreased expression of xylosyltransferase I, galactosyltransferase I, *N*-acetylglucosaminyl- and glucuronosyltransferase, or *N*-sulfotransferase I of these cells as expected (Fig 5B, left). By contrast, binding of mPNA(H)-Fc to each of the proteoglycan-deficient CHO mutant cells was approximately half of its binding to wild-type CHO cell (Fig 5B, right).

## Discussion

In this study, we established mutated PNA (mPNA(H)) that could bind to heparin by screening of mutated PNA library displayed on the surface of mammalian cells [6]. Substitution of only six amino acids in PNA loop C significantly affected the lectin's sugar-binding specificity from Galβ1-3GalNAc to heparin, whereas the other 244 amino acid residues within this 250 residue protein were completely identical (Fig 1B). Interestingly, mutated PNA with an affinity for heparin contained the heparin-binding like motif Arg-His-X-X-Arg-Arg-X-X in carbohydrate-binding loop C (Fig 1B). Heparin-binding motifs such as XBBXBX and XBBBXXBX (B: Arg, Lys, His, X: non-basic residues) have been identified in a variety of heparin-binding proteins [8,19]. Basic amino acid residues in heparin-binding motif could interact with sulfate groups and/or carboxy groups on GAG disaccharide-repeating units consisting of an amino sugar (*N*-acetylglucosamine, *N*-acetylgalactosamine) and an uronic acid (glucuronic acid, iduronic acid). An alanine-substitution experiment using mPNA(H)-Fc indicated that three Arg residues in loop C were largely involved in binding to heparin (Fig 4B) and that this heparin-binding like motif was specific for heparin but not for 2-*O*-desulfated, 6-*O*-desulfated, or *N*-desulfated heparins (Fig 4A). However, because dissociation constant of the interaction between mutated PNA and heparin was 2.47 x 10^−8^ M (Table 1), partially desulfated heparins might be still be good ligands for mutated PNA. In inhibition assay of mutated PNA(H)-Fc binding to heparin, dextran sulfate inhibited mPNA(H)-Fc binding to heparin-BSA at lower concentrations that heparin (Fig 5A). This suggests that the carboxy group of glucuronic/iduronic acids was not critical for heparin binding, as well as that mPNA(H)-Fc can accommodate sulfate groups with different topologies. Binding of mutated PNA-Fc to the proteoglycan-deficient cells was approximately half of that to wild-type CHO cell. These proteoglycan-deficient cells were established from CHO cells treated with mutagen (ethyl methanesulfonate, EMS) and screened by decreased incorporation of ^35^SO_4_ in the cells [15]. Based on the reports about proteoglycan-deficient cell lines [7,15–18], most of proteoglycan-deficient cells showed the decreased synthesis of GAGs and glycosyltransferase activity involved in GAG biosynthesis was diminished. However, the activity of a mutated glycosyltransferase is not disappeared completely. This may be the reason why mutated PNA-Fc partially bound to GAG-deficient CHO cell lines.

GAGs display heterogeneity with regard to the position and extent of sulfation of their sugar residues, and C5 epimerization of uronic acids. Such differences in sulfated position and epimerization in disaccharide units play an essential role in the interaction between GAGs and GAG- and heparin-binding proteins such as growth factors, cytokines, chemokines, extracellular matrix proteins, coagulation factors and lipid metabolic enzymes [8,20,21].

Lectins contain a sugar-binding scaffold and are useful probes for monitoring glycan structures expressed on cell surfaces and attached to proteins, since they can distinguish between different sugar sequences within complex glycan structures. In this report, we demonstrated that the heparin-binding like motif on loop C of leguminous lectin could confer heparin-binding affinity to the scaffold of these proteins. Our approach could allow us to obtain additional mutated PNAs with preference for other kinds of glycosaminoglycans such as 2-*O*-desulfated, 6-*O*-desulfated, or *N*-desulfated heparin. Such engineered PNAs would provide strong probes for the analysis of the biological significance of sulfated heparan sulfate and heparin, since there are few or no antibodies or lectins that can clearly distinguish between GAG chains with different position and extent of sulfation.

## Supporting Information

S1 Fig Structures of oligosaccharides and glycoproteins used for the glycan microarray analysis. Structures of polyacrylamide-based oligosaccharides and glycoproteins used for glycan array analysis. Symbols corresponding to each monosaccharide are shown in the panel. Thin and thick bars represent alpha- and beta-linkages, respectively. Glycosidic linkage positions are shown by the numbers on the right side of the panel.
(TIF)

## Abbreviations

GAG: glycosaminoglycan
PNA: peanut agglutinin
Gal: galactose
GalNAc: *N*-acetylgalactosamine
NeuAc: *N*-acetylneuraminic acid
HP: heparin
CS: chondroitin sulfate
HA: hyaluronic acid
CH: chondroitin
HS: heparan sulfate
MFI: mean fluorescent intensity
SPR: surface plasmon resonance
ELISA: enzyme-linked immunosorbent assay
FACS: fluorescence-activated cell sorter
FBS: fetal bovine serum
GFP: green fluorescent protein
PCR: polymerase chain reaction
CHO: Chinese hamster ovary
RU: resonance unit
