# Supplementary figures and images for: Mutated Leguminous Lectin Containing a Heparin-Binding like Motif in a Carbohydrate-Binding Loop Specifically Binds to Heparin
Source: PLoS One. 2015 Dec 29;10(12):e0145834. doi: 10.1371/journal.pone.0145834 (PMC4701002; doi:10.1371/journal.pone.0145834)

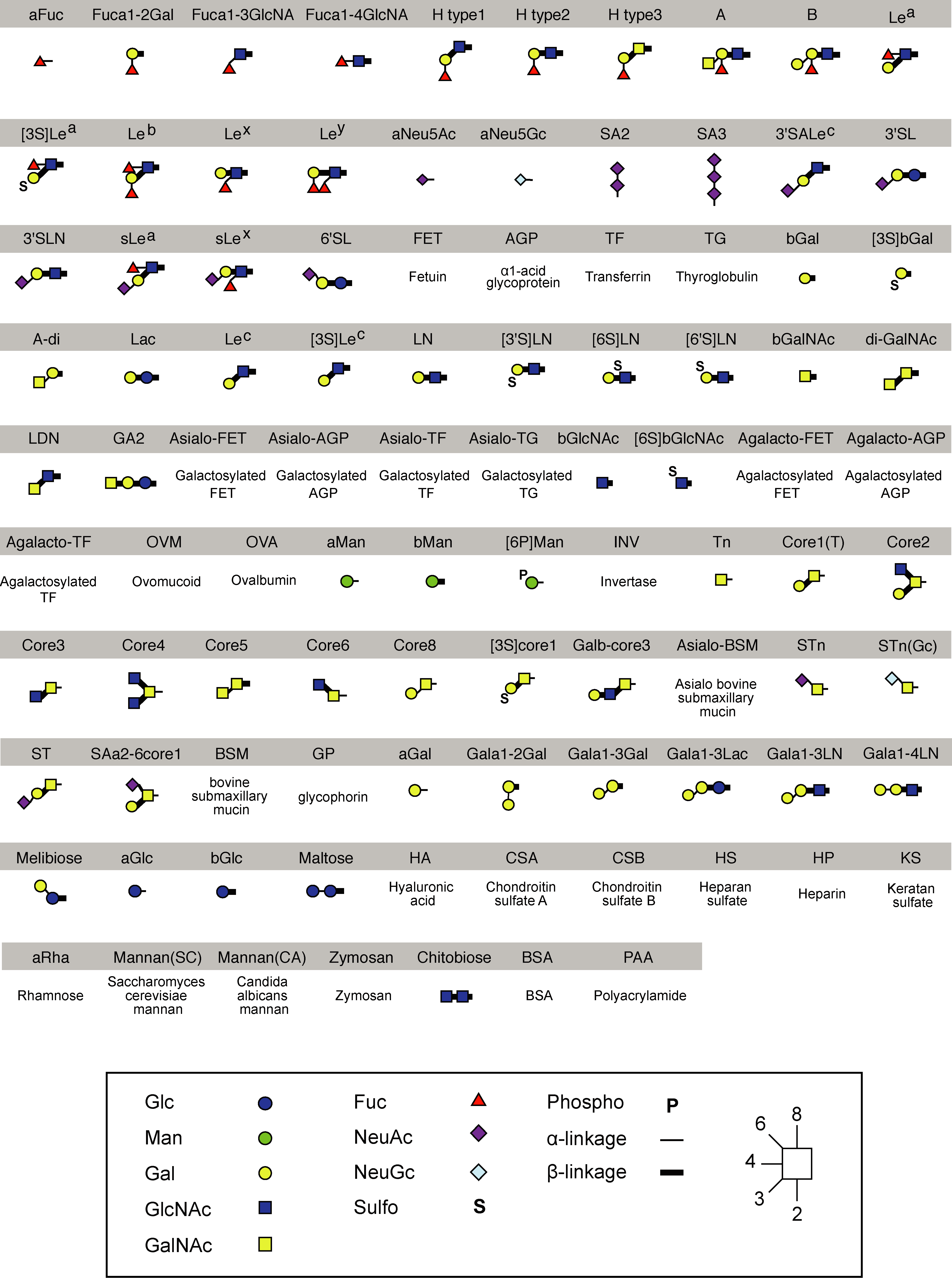

Supplement: S1 Fig — Structures of polyacrylamide-based oligosaccharides and glycoproteins used for glycan array analysis. Symbols corresponding to each monosaccharide are shown in the panel. Thin and thick bars represent alpha- and beta-linkages, respectively. Glycosidic linkage positions are shown by the numbers on the right side of the panel. (TIF) [file pone.0145834.s001.tif]
